# Low-Dose Apatinib Combined With S-1 in Refractory Metastatic Colorectal Cancer: A Phase 2, Multicenter, Single-Arm, Prospective Study

**DOI:** 10.3389/fonc.2021.728854

**Published:** 2021-09-02

**Authors:** Ning Li, Wenying Deng, Guifang Zhang, Yali Du, Yanwei Guo, Yijie Ma, Chen Wei, Liangyu Bie, Chi Zhang, Tao Song, Suxia Luo, Baijun Fang

**Affiliations:** ^1^Department of Medical Oncology, Henan Cancer Hospital, Affiliated Cancer Hospital of Zhengzhou University, Zhengzhou, China; ^2^Department of Medical Oncology, Xinxiang Central Hospital, Xixiang, China; ^3^Department of Medical Oncology, Fifth Affiliated Hospital of Zhengzhou University, Zhengzhou, China; ^4^Department of Medical Imaging, Henan Cancer Hospital, Affiliated Cancer Hospital of Zhengzhou University, Zhengzhou, China; ^5^Department of Hematology, Henan Cancer Hospital, Affiliated Cancer Hospital of Zhengzhou University, Zhengzhou, China

**Keywords:** apatinib, S-1, colorectal cancer, metastatic, efficacy

## Abstract

**Purpose:**

Apatinib is an approved third-line treatment for metastatic gastric cancer in China and demonstrates good safety, tolerability, and efficacy in other advanced solid tumors. The aim of this prospective, single-arm, multicenter, phase 2 study was to assess the efficacy and safety of low-dose apatinib combined with S-1 in the treatment of refractory mCRC.

**Patients and Methods:**

Patients with refractory mCRC were enrolled and administered apatinib combined with S-1 until disease progression, patient decision to withdraw, or unacceptable toxic effects. The primary endpoint was investigator-evaluated progression-free survival (PFS) and the secondary endpoints were overall survival (OS), objective response rate (ORR), disease control rate (DCR).

**Results:**

From December 2017 to December 2018, 30 patients were enrolled and 29 patients were eligible for the evaluation of efficacy and safety. The median PFS (mPFS) and OS (mOS) were 7.9 and 12.9 months, respectively. Exploratory analysis revealed that patients administered S-1 ≥ 70 days achieved longer mPFS and mOS. Four patients achieved a partial response, 22 achieved stable disease, and three had progressive disease, attributing to an ORR of 13.79% and a DCR of 89.66%. Ten grade 3 adverse events were reported and the frequency of each grade 3 adverse event was less than 5%. No grade 4 side events were observed.

**Conclusions:**

These results indicated that apatinib combined with S-1 showed promising efficacy and manageable toxicity in patients with progressive mCRC after at least 2 prior lines of therapy, making it a promising therapeutic option for mCRC treatment.

**Clinical Trial Registration:**

https://clinicaltrials.gov/ct2/show/NCT03397199, identifier NCT03397199.

## Introduction

Colorectal cancer (CRC) is the third most commonly diagnosed cancer and the second leading cause of cancer deaths worldwide (1). According to the 2015 Chinese Cancer Statistics, the incidence and mortality of CRC increased (2). Surgical resection and preoperative chemoradiotherapy are radical measures for patients in the early or locally advanced stages (3). However, approximately one-quarter of patients present with metastatic disease at diagnosis, and another quarter eventually develop metastases during the course of their treatment. Systemic chemotherapy regimens (fluorouracil with leucovorin in addition to either irinotecan or oxaliplatin) remain the standard treatment for most patients with metastatic CRC (mCRC) (4). Chemotherapy in combination with agents targeting the vascular endothelial growth factor (VEGF) pathway or the epidermal growth factor receptor (EGFR) also improve outcomes for mCRC (5). In addition to third-line treatment options for Chinese patients with mCRC including trimethoprim fluorouracil (TAS-102) and regorafenib, treatment options are still limited for patients with mCRC who are refractory to standard first- and second-line therapy. Therefore, developing third-line therapeutic strategies for mCRC is necessary, especially in China.

Angiogenesis, mainly regulated by the VEGF pathway, plays important roles in the development of colorectal cancer, and anti-angiogenesis has been an important treatment strategy for patients with mCRC (6). For instance, bevacizumab, anti-VEGF antibody drug, has been used to treat mCRC in both first- and second-line settings in combination with chemotherapy (7, 8). Regorafenib and fruquintinib, as small-molecule multi-receptor tyrosine kinase inhibitors, are recommended as third-line therapies, however, their benefits are only modestly improved and adverse effects might be difficult to manage (9–11).

Apatinib, a novel small-molecule tyrosine kinase inhibitor (TKI), which selectively binds to and strongly inhibits vascular epidermal growth factor receptor 2 (VEGFR2), has shown efficacy in various types of cancers (12). A Phase III study showed that apatinib improves overall survival (OS) and progression-free survival (PFS) of patients with heavily pretreated gastric cancer (13) and it was approved as a subsequent-line therapy for gastric cancer by the China State Food and Drug Administration. Furthermore, several phase II/III trials have proven its satisfactory efficacy and tolerable adverse events in many other cancer types, such as metastatic triple-negative breast cancer (14), non-small-cell lung cancer (15), and ovarian cancer (16). Recently, two prospective studies reported that apatinib monotherapy in a third-line setting displays promising activities in patients with refractory colorectal cancer (17, 18). Further, several retrospective studies evaluated the safety and efficacy of apatinib in patients with heavily treated mCRC and suggested that apatinib is effective as a third-line treatment (19–21).

The oral fluoropyrimidine S-1, as a prodrug of 5-fluorouracil (5-FU), has exhibited comparable efficacy results compared to 5-FU and capecitabine in mCRC (22–24), with a lower incidence of the hand-foot syndrome and reduced gastrointestinal toxicities (23, 25). Previous studies showed S-1 combined with irinotecan to have an equal effect as FOLFIRI as second-line chemotherapy for mCRC (24).

Based on these studies, here, we aimed to report the efficacy and safety of apatinib combined with S1 in patients with mCRC after the failure of at least two lines of previous treatments.

## Materials and Methods

### Patients

This investigator-initiated, multicenter, prospective phase 2 study was open to patients with refractory metastatic colorectal cancer. Between December 2017 and December 2018, a total of 30 patients from three oncology centers in China were enrolled in this trial.

The criteria for patient inclusion were as follows: (1) Aged 18-75 years, diagnosed with mCRC, and received more than two lines of standard chemotherapy (irinotecan and oxaliplatin). 20 patients previously treated with targeted therapy. The targeted therapy drugs were bevacizumab in 19 patients (65%), cetuximab in 2 patients (7%); (2) an Eastern Cooperative Oncology Group performance status (ECOG PS) of 0 to 1; (3) at least one measurable lesion evaluated according to the Response Evaluation Criteria in Solid Tumors (RESIST) criteria (1.1); (4) absolute neutrophil count ≥ 1.5× 10^9^ cells per L, and platelet count ≥ 80 × 10^9^ cells per L; Key exclusion criteria were; previous exposure to apatinib, allergies to any of the excipients, uncontrolled blood pressure, any history of surgery or gastrointestinal perforation, and bleeding or obstruction within 28 days before day 1 of the enrollment. Patients with a history of any one or more of the following conditions within the past 6 months before day 1 of this study were also excluded; central nervous system metastasis, myocardial infarction, unstable angina, and congestive heart failure.

Study procedures were approved by the Research Ethics Board of Henan Cancer Hospital and were following the Declaration of Helsinki. All patients provided written informed consent before enrollment in the study.

### Study Design and Treatment

The study was a single-arm, multicenter, phase II clinical trial. This study aimed to assess the safety and efficacy of apatinib combined with S-1 in the treatment of mCRC patients. All patients received oral apatinib (HengRui Medicine Co., Ltd., China) 250 mg daily, combined with S1 (40 mg for BSA < 1.2, 50 mg for 1.2 < BSA < 1.5, or 60 mg for BSA >1.5; between days 1-14, for 3 weeks) until disease progression or intolerable toxic effects. If intolerable toxic effects were observed (hypertension, hand and foot symptom, albuminuria), apatinib was temporarily discontinued until the patient had recovered from toxicity. If side effects were caused by S-1, the dose was reduced. If toxicity abated with the dose reduction, the dose of S-1 would not return to the initial dose. Regardless of permanent discontinuation of Apatinib or S-1, patients continued monotherapy with another drug. Patients who completed the first 28 days of treatment were evaluated for efficacy and re-evaluate every 56 days. The chest/abdominal/pelvic CT scan and traditional biomarkers [carcinoembryonic antigen (CEA), cancer antigen 72-4 (CA724), and CA199] were rechecked for disease assessment. The CT images were assessed by professional radiologists who have been involved in the evaluation of several clinical studies at our center, and who were blinded to the therapeutic outcomes and clinicopathological features.

### Outcomes

The primary analysis endpoint was PFS and secondary analysis endpoints were disease control rate (DCR), objective response rate (ORR), and OS. PFS was calculated from the date of oral treatment to the date of disease progression, death, or the last follow-up. OS was determined as the period between the date of oral treatment and the date of death or the last follow-up. Tumor response was evaluated by radiologists and oncologists according to RECIST version 1.1 as complete response (CR), partial response (PR), stable disease (SD), and progressive disease (PD). DCR was defined as the percentage of patients with CR, PR, and SD. Adverse events were reviewed and classified from patients’ medical history and telephone follow-up according to the National Cancer Institute Common Toxicity Criteria for Adverse Events version 5.0 (CTCAE 5.0).

### Statistical Analysis

PASS 15 software and a single-sample log-rank test were used to calculate the sample size. Given that the expected median PFS (mPFS) of 5.5 months with 80% power, α=0.05 (two-sided) and β=0.20, an enrollment period of 24 months, and a follow-up period of 6 months, we estimated that at least 29 subjects needed to be enrolled. PFS was defined as the time from the start of low-dose apatinib combined with S-1 therapy to the first disease progression or death from any cause. OS was evaluated as the time from the start of low-dose apatinib combined with S-1 therapy to death from any cause.

All data were statistically analyzed using the Statistical Package for the Social Sciences (SPSS) 22.0 software (SPSS Inc. Chicago IL, USA). The Kaplan-Meier method and the log rank test were used to analyze the OS and PFS. Multivariate analyses of cox regression model were applied to evaluate the effects of multiple covariates on survival.

## Results

### Patient Characteristics

Of the 32 patients screened, 30 were enrolled in the study and one was excluded because of failure to follow-up. The main clinical features of the 29 patients are shown in **Table 1**. The median age of the patients was 53 (range 25-73) years, while 17 males and 12 females were included in this study. The proportion of patients with ECOG PS scores of 0 and 1 were 3.4% (1/29) and 96.6% (28/29), respectively. (5) All patients were tested for KRAS, BRAF V600E, and MSI status. We identified 8 patients (28%) with KRAS mutations. All patients with BRAF V600E wild type and MSS status were detected. The anti-VEGF agent used was bevacizumab in 19 patients (65%) prior to the use of apatinib. 16 patients received apatinib as the third-line treatment, 13 patients received apatinib as the fourth- or higher-line therapy. Eleven patients were diagnosed with primary colon adenocarcinoma and 18 patients with rectal cancer. Most patients (23/29, 79.3%) were tested for *KRAS* mutation.

**Table 1:** Baseline characteristics of 29 patients enrolled in the clinical study.

Characteristic	Value
ECOG,n(%)	
0	1 (3.4)
1	28 (96.6)
Sex,n (%)	
Male	17 (58.6)
Female	12 (41.4)
Age,median (range),y	53 (25-73)
Location,n (%)	
right	2 (6.9)
left	27 (93.1)
Line of apatinib,n (%)	
3 line	16 (55.2)
further line	13 (44.8)
S-1 dosage (mg),n (%)	
40	18 (62.1)
60	11 (37.9)
Apatinib time,median (range),d	125 (21-330)
S-1 time,median(range),d	60 (14-165)
KRAS type,n (%)	
KRAS mutation	15 (51.7)
KRAS wild	8 (27.6)
No test	6 (20.7)

### Efficacy

**Figure 1:**
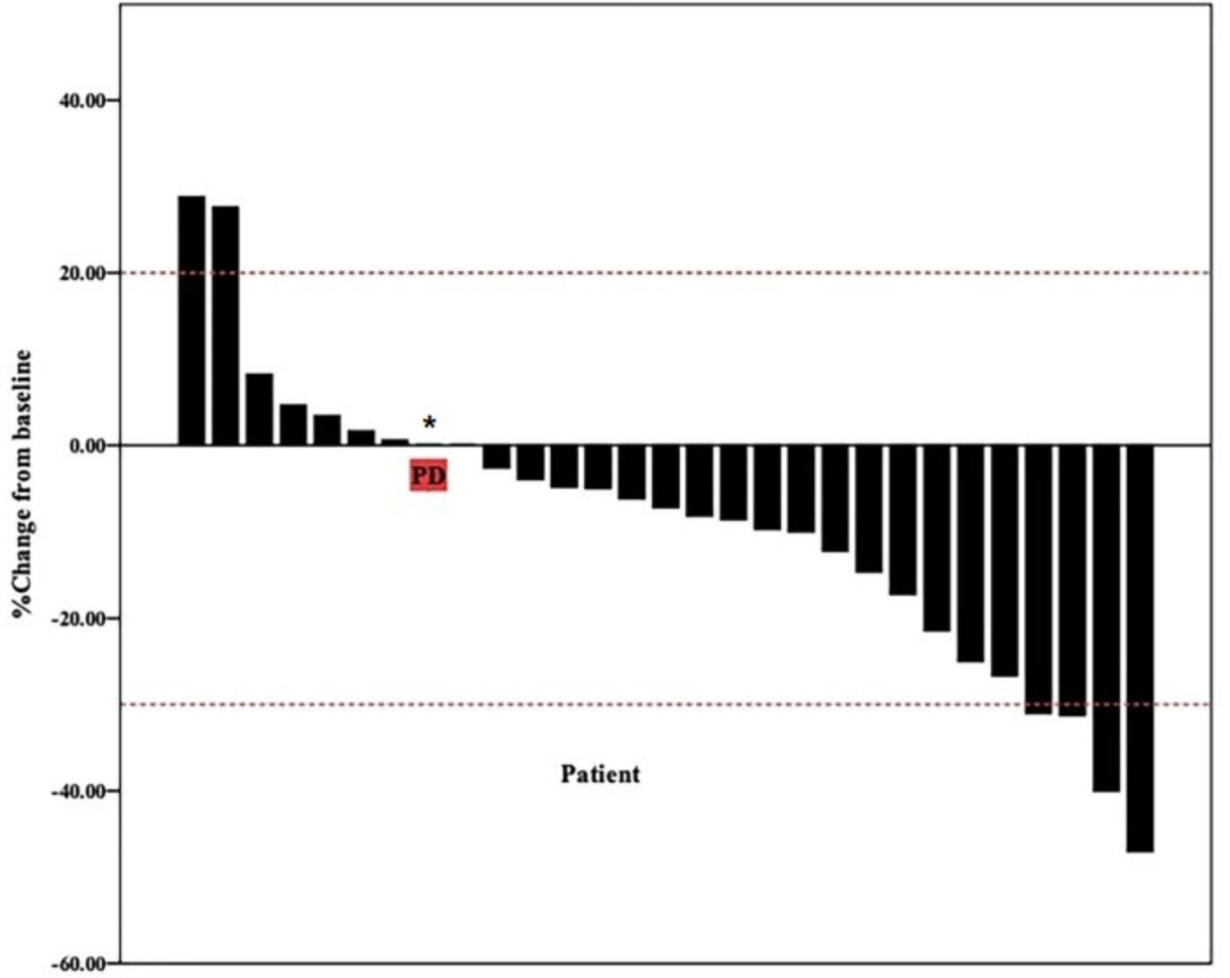
Waterfall plot of best reduction in tumor size compared with baseline. Bar graph depicting the best percentage change in tumor size compared to baseline in 29 patients. * patient evaluation of tumor is SD, but pleural effusion increased significantly.

**Figure 2:**
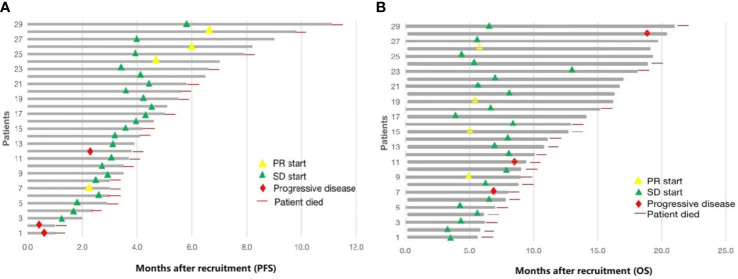
Summary of 29 patients treated with low-dose apatinib and S1. **(A)** progression-free survival (PFS). **(B)** overall survival (OS).

**Figure 3:**
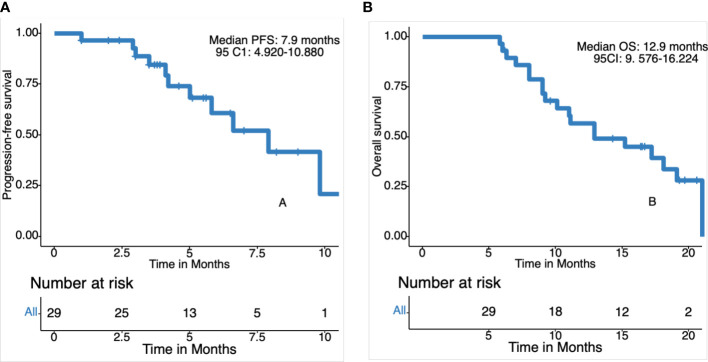
Kaplan-Meier curves for progression-free survival (PFS) and overall survival (OS). **(A)** Progression free survival in 29 patients. **(B)** Overall survival in 29 patients.

The date cut-off date for the analyses was January 30, 2021. At the time of analysis 29 events were observed. Total of 29 patients was evaluated for therapeutic efficacy and adverse events after taking apatinib combined with S1. The median duration of treatment was 4.6 months. Among all 29 patients, no patient achieved CR, four patients achieved PR, and 22 patients were SD. The waterfall plots of evaluable patients showing the best change compared with the baseline are shown in **Figure 1** and the treatment response is shown in **Figure 2**. An ORR of 13.79% and a DCR of 89.66% were achieved, and two patients developed a cavity in metastatic lung disease. Median PFS was 7.9 months (95% confidence interval [CI]: 4.92-10.88) and median OS (mOS) was 12.9 months (95% CI: 9.576-16.224). Kaplan-Meier curves for PFS and OS are shown in **Figure 3**.

Subgroups analysis showed that a longer mPFS and mOS were observed in patients who received S-1 treatment time≥ 70 days, compared to those who received S-1 treatment time< 70 days (9.8 vs 4.2 months, *p*=0.001; 19.1 vs 9.2 months, *p*=0.008). Patients with a lower neutrophil-to-lymphocyte ratio also exhibited better mPFS and mOS than those with higher neutrophil-to-lymphocyte ratio (5.6 vs 3.5 months, *p*=0.006; 19.1 vs 8 months, *p*=0.005). Low D-dimer was associated with a better OS than a high one (15.2 vs 8 months, *p*=0.033). Pre-treatment with or without targeted therapy did not affect survival. Exploratory subgroup analysis is shown in **Table 2**. The Kaplan-Meier curves for PFS and OS in different subgroups are shown in **Supplementary Figures 4**, **5**. Univariate analysis showed that the D-dimer, S-1 treatment, and neutrophil-to-lymphocyte ratio were significantly associated with OS (hazard ratio [HR], 3.23, 0.25, and 4.22, respectively; **Supplementary Table 1**). We then performed Cox multivariate regression analysis. Only the neutrophil-to-lymphocyte ratio remained significantly associated with OS (HR, 2.71; **Supplementary Table 1**).

**Table 2:** Exploratory subgroup analyses of all patients.

Variable	cases	median PFS (95%CI)	P-value	median OS (95%CI)	P-value
Total patients	29	7.9 months (4.920-10.880)		12.9 months (9.576-16.224)	
**Age**					
<60	20	9.8 (1.717-17.883)	0.144	15.200 (6.654-23.746)	0.078
≥60	9	5.8 (3.801-7.799)		9.000 (7.539-10.461)	
**Gender**					
Male	17	6.6 (2.314-10.886)	0.216	10.100 (7.545-12.655)	0.100
Female	12	9.8 (3.769-15.831)		19.100 (14.897-23.303)	
**Line of apatinib**					
3 line	16	6.6 (4.724-8.476)	0.588	10.100 (6.572-13.628)	0.502
further line	13	9.8		15.200 (8.383-22.017)	
**Location**					
right	2	4.2	0.226	6.000	0.082
left	27	7.9 (4.571-11.229)		12.900 (6.133-19.667)	
**S-1 dosage (mg)**					
40	18	5.8 (3.648-7.952)	0.147	10.100 (7.190-13.010)	0.103
60	11	NE		18.100 (12.747-23.453)	
**Apatinib time (days)**					
≥140	16	9.8 (6.930-12.670)	0.001	18.100 (10.489-25.711)	0.134
<140	13	4.1 (3.478-4.722)		9.600 (6.072-13.128)	
**S-1 time (days)**					
≥70	13	9.8 (6.936-12.664)	0.001	19.100 (17.669-20.531)	0.008
<70	16	4.2 (3.346-5.054)		9.200 (8.024-10.376)	
**Hypertension symptom**					
Yes	6	NE	0.361	15.200 (4.224-26.176)	0.507
No	23	6.6 (4.369-8.831)		11.100 (7.813-14.387)	
KRAS type^*^					
KRAS mutation	14	9.800	0.383	19.100	0.458
KRAS wild	8	NE		12.900 (5.383-20.417)	
**D-dimer (mg/L) #**					
≥1.30	7	5.800	0.112	8.000 (3.637-12.363)	0.033
<1.30	18	6.600		15.200	
**Neutrophil: Platelets ※**					
≥0.018	13	6.600 (3.028-10.172)	0.306	10.100 (7.869-12.331)	0.490
<0.018	15	7.900 (4.305-11.495)		18.100 (6.825-29.375)	
**Neutrophil : Lymphocyte #**					
≥2.75	7	4.100 (3.051-5.149)	0.006	8.000 (5.434-10.566)	0.005
<2.75	18	NE		19.100 (8.296-29.904)	
**Anti-angiogenic drug use**					
Bevacizumab	19	9.800	0.915	12.900 (5.938-19.862)	0.881
No Bevacizumab	10	7.900 (3.811-11.989)		11.000 (7.746-14.254)	

*Data available for 22 patients.

**#**Data available for 25 patients.

**※**Data available for 28 patients. NE, Not Estimable.

### Safety

Total of 29 patients was eligible for the safety analysis. And 28 (96.6%) patients experienced adverse events during treatment and the incidence of different adverse events is summarized in **Table 3**. The most common adverse events at all grades were thrombocytopenia (37.93%), followed by leukopenia and elevated transaminase with same incidence rates of 27.59%. A total of ten grade 3 adverse events were reported and the frequency of each grade 3 adverse event was less than 5%. Grade 4 toxicities did not occur in any of the patients. No serious hepatic or renal function impairment was reported and no toxicity-related deaths occurred during the study.

**Table 3:** Summary of treatment-related adverse reactions.

	Any grade n (%)	Grade 1 n (%)	Grade 2 n (%)	Grade 3 n (%)	Grade 4 n (%)
Clinical adverse event					
Fatigue	4 (13.79%)	4 (13.79%)	0	0	0
Hand-foot skin reaction	1 (3.45%)	1 (3.45%)	0	0	0
Diarrhea	1 (3.45%)	1 (3.45%)	0	0	0
Voice changes	1 (3.45%)	1 (3.45%)	0	0	0
Hypertension	6 (20.69%)	1 (3.45%)	4 (13.79%)	1 (3.45%)	0
Cholecystitis	2 (6.90%)	2 (6.90%)	0	0	0
Nausea	3 (10.34%)	3 (10.34%)	0	0	0
Weight loss	1 (3.45%)	1 (3.45%)	0	0	0
Dizziness	1 (3.45%)	1 (3.45%)	0	0	0
Toothache	1 (3.45%)	1 (3.45%)	0	0	0
Stoma infection	1 (3.45%)	1 (3.45%)	0	0	0
Laboratory abnormalities					
Thrombocytopenia	11 (37.93%)	7 (24.14%)	3 (10.34%)	1 (3.45%)	0
Anemia	3 (10.34%)	1 (3.45%)	1 (3.45%)	1 (3.45%)	0
Leukopenia	8 (27.59%)	4 (13.79%)	4 (13.79%)	0	0
Neutropenia	6 (20.69%)	2 (6.90%)	4 (13.79%)	0	0
Hyperbilirubinemia	16 (%)	8 (27.59%)	7 (24.14%)	1 (3.45%)	0
Hyperbiliary acidemia	2 (6.90%)	2 (6.90%)	0	0	0
Hypertriglyceridemia	3 (10.34%)	3 (10.34%)	0	0	0
Hypercholesterolemia	1 (3.45%)	1 (3.45%)	0	0	0
Elevated transaminase	8 (27.59%)	6 (20.69%)	1 (3.45%)	1 (3.45%)	0
Elevated lactate dehydrogenase	1 (3.45%)	1 (3.45%)	0	0	0
Hypoalbuminemia	2 (6.90%)	2 (6.90%)	0	0	0
Hyponatremia	1 (3.45%)	0	0	1 (3.45%)	0
Hypokalemia	2 (6.90%)	1 (3.45%)	0	1 (3.45%)	0
Hyperkalemia	1 (3.45%)	1 (3.45%)	0	0	0
Hypocalcemia	3 (10.34%)	0	3 (10.34%)	0	0
Hypophosphatemia	1 (3.45%)	0	0	0	0
Urinary occult blood	2 (6.90%)	1 (3.45%)	0	1 (3.45%)	0
Proteinuria	2 (6.90%)	1 (3.45%)	0	1 (3.45%)	0
Urine leukocytes	1 (3.45%)	0	0	1 (3.45%)	0

Data were available for 29 patients with advanced colorectal cancer after using apatinib and S-1.

The most common apatinib-related adverse events were hypertension (20.69%), proteinuria (6.90%), and fatigue (13.79%). Overall, 5 (17.24%) of the 29 patients had drug-related grade 3 adverse events, and 2 (6.90%) experienced treatment modifications because of apatinib-related proteinuria.

Anemia and thrombocytopenia developed in 10.34 and 37.93% of the patients, respectively. One patient had grade 3 thrombocytopenia, and one patient, who experienced grade 3 anemia, received a red blood cell transfusion.

The most common nonhematological toxicities were fatigue, nausea, and elevated transaminase. Among them, the incidence of grade 1-2 fatigue, nausea, and elevated transaminase were 13.79, 10.34, and 24.14%, respectively. Only one patient had grade 3 elevated transaminase.

## Discussion

To the best of our knowledge, this is the first prospective study to evaluate the efficacy and safety of combination therapy with apatinib, an oral agent with anti-angiogenic properties, and S1 in patients with chemo refractory mCRC. Promising results were observed using this treatment regimen. More than half of the patients had been treated with chemotherapy and targeted therapy and their prognosis was very poor because of the absence of effective regimens. However, the patients co-treated with apatinib and S1 achieved an unexpected objective response and the DCR. More importantly, mPFS and mOS were also promising. Furthermore, the combination of apatinib and S-1 had manageable toxicity. Consequently, patients with mCRC, who had progressed disease after being exposed to at least 2 prior lines of therapy, could benefit from the combination therapy with apatinib and S1.

S-1 showed superiority in terms of cost-effectiveness and patient preference. A phase II trial reported an ORR of 14.3% and a PFS of 3.03 months for patients with mCRC who were treated with oral S-1 monotherapy as a third-line therapy (26). In addition, apatinib as a third-line monotherapy in patients with mCRC achieved an ORR of 8.3-13.9%, an mPFS of 3.7-4.8 months, and an mOS of 7.3-9.1 months (17–21). Although cross-trial comparisons were tough, our study presented that the mPFS and mOS of apatinib combined with S-1 were superior and the ORR was similar to those observed in previous studies.

Numerous patients with mCRC having disease progression after receiving all standard therapies still exhibit an adequate ECOG PS to tolerate further treatment. Recent drugs regorafenib, fruquintinib, and TAS-102 have superior efficacy compared with the placebo, therefore they would be suitable for third-line therapy in mCRC (11, 27). Unfortunately, the survival effect of the third-line CRC treatment in phase 3 trials is modest. In the CORRECT trial, regorafenib monotherapy yielded an mOS of 6.4 months and an mPFS of 1.9 months, which was also validated in an Asian population in the CONCUR trial (PFS: 3.2 months; OS: 8.8 months) (9, 10). The phase III FRESCO trial of fruquintinib monotherapy reported an ORR of 4.7%, an mOS of 9.3 months, and an mPFS of 3.7 months (11). In the RECOURSE trial, TAS-102 monotherapy achieved an ORR of 1.6%, a DCR of 44%, an mOS of 7.1 months, and an mPFS of 2.0 months (28). In the trial that tested TAS-102 with or without bevacizumab in patients with chemo refractory mCRC, an ORR of 2.1%, an mOS of 9.6 months, and an mPFS of 4.6 months were reported (29). In our study, we observed an ORR of 13.79%, an mPFS of 7.9 months, and an mOS of 12.9 months. Compared to the patients included in our clinical trials, approximately 50% of patients in the RECOURSE and CORRECT trials were treated in 4 or more lines. Third-line or post-third-line treatment may increase OS expectancy in patients of mCRC.

The safety profile of available third-line/further-line mCRC therapies may help treatment selection for patients when the quality of life is threatened. In this study, there were no grade 4 adverse events or treatment-related deaths and no grade 3/4 neutropenia occurred in any patients. In contrast, grade 3 adverse events were observed in almost 50% of the patients administered regorafenib (mostly hand-foot skin reaction, fatigue, diarrhea, hypertension, and rash) (9). Cetuximab and panitumumab cause almost 30% of grade 3-4 toxicities (mainly rash and dermatitis acneiform) in patients (30). In the study conducted by Li et al., the most frequently observed drug-related adverse events were hypertension (69.5%), proteinuria (47.8%), and hand-foot syndrome (45.6%) (31). In another study, the incidences of the three most common grade 3/4 adverse events were higher than those in our study (hypertension, 7%; hand-foot syndrome, 6%; proteinuria, 4%) (32). In the present study, most adverse events were of grades 1-2 and were well tolerated and controlled, maybe because we used low-dose apatinib.

It must be pointed out that exploratory analysis revealed that patients that received S-1 treatment ≥ 70 days achieved longer mPFS and mOS, suggesting that patients obtained extended survival benefits. Additionally, patients with hypertension showed a tendency of prolonged PFS and OS which was consistent with the finding that anti-angiogenic adverse events could be predictive biomarkers for treatment efficacy (33).

We acknowledge that this study had some limitations. This was a single-arm study without a control group for comparison, and thus selection bias could not be excluded due to the non-randomized design. Considering that fewer adverse events depend on administering low-doses of apatinib, further studies are needed to validate our results. The phase III trial of low-dose apatinib with S1 is ongoing and good results are expected.

## Conclusions

In conclusion, this study is the first to report a combination therapy with apatinib and S1 in patients with refractory mCRC. We expect apatinib plus S-1 to demonstrate promising efficacy and manageable toxicity as a third-line therapy for mCRC, which warrants further investigation.

## Data Availability Statement

The raw data supporting the conclusions of this article will be made available by the authors, without undue reservation.

## Ethics Statement

The Research Ethics Board of Henan Cancer Hospital approved the protocol and the study was in accordance with the Declaration of Helsinki. The patients/participants provided their written informed consent to participate in this study. Written informed consent was obtained from the individual(s) for the publication of any potentially identifiable images or data included in this article.

## Author Contributions

Conception and design: NL, SL, and BF. Development of methodology: WD. Acquisition of data (provided acquired and managed patients, provided facilities, etc.): GZ, YD, YG, and YM. Analysis and interpretation of data (e.g., statistical analysis, biostatistics, computational analysis): CW. Writing, review, and/or revision of the manuscript: LB. Administrative, technical, or material support (i.e., reporting or organizing data, constructing databases): CZ. Study supervision: TS. All authors contributed to the article and approved the submitted version.

## Funding

Key Scientific and Technological Projects in Henan Province (202102310111); Key projects of Henan Provincial Department of Education (13A320440). Joint Funds of the National Natural Science Foundation of China (U2004132)

## Conflict of Interest

The authors declare that the research was conducted in the absence of any commercial or financial relationships that could be construed as a potential conflict of interest.

## Publisher’s Note

All claims expressed in this article are solely those of the authors and do not necessarily represent those of their affiliated organizations, or those of the publisher, the editors and the reviewers. Any product that may be evaluated in this article, or claim that may be made by its manufacturer, is not guaranteed or endorsed by the publisher.

## Supplementary Material

Supplementary Table 1 Univariate and Multivariate Cox analyses of the S1 time, D dimer and NLR.
