# Supplementary material for: Low-Dose Apatinib Combined With S-1 in Refractory Metastatic Colorectal Cancer: A Phase 2, Multicenter, Single-Arm, Prospective Study
Source: Front Oncol. 2021 Sep 2;11:728854. doi: 10.3389/fonc.2021.728854 (PMC8443771; doi:10.3389/fonc.2021.728854)
Supplement: Supplementary Table 1 — Univariate and Multivariate Cox analyses of the S1 time, D dimer and NLR. [file Table_1.docx]

**Supplementary Table 1. Univariate and Multivariate Cox analyses of the S1 time, D dimer and NLR.**

| ﻿Variable | ﻿Univariate COX  HR ﻿95% CI | *P* | Multivariate COX  HR ﻿95% CI | *P* |
| --- | --- | --- | --- | --- |
| S1 time | 0.25 (0.09-0.71) | 0.009 | 0.24 [0.05, 1.08] | 0.062 |
| D dimer | 3.23 (1.04-10.08) | 0.043 | 2.07 [0.57, 7.51] | 0.267 |
| NLR | 4.22 (1.44-12.39) | 0.009 | 2.71 [1.37, 6.94] | 0.021 |
