# Supplementary figures and images for: Low-Dose Apatinib Combined With S-1 in Refractory Metastatic Colorectal Cancer: A Phase 2, Multicenter, Single-Arm, Prospective Study
Source: Front Oncol. 2021 Sep 2;11:728854. doi: 10.3389/fonc.2021.728854 (PMC8443771; doi:10.3389/fonc.2021.728854)

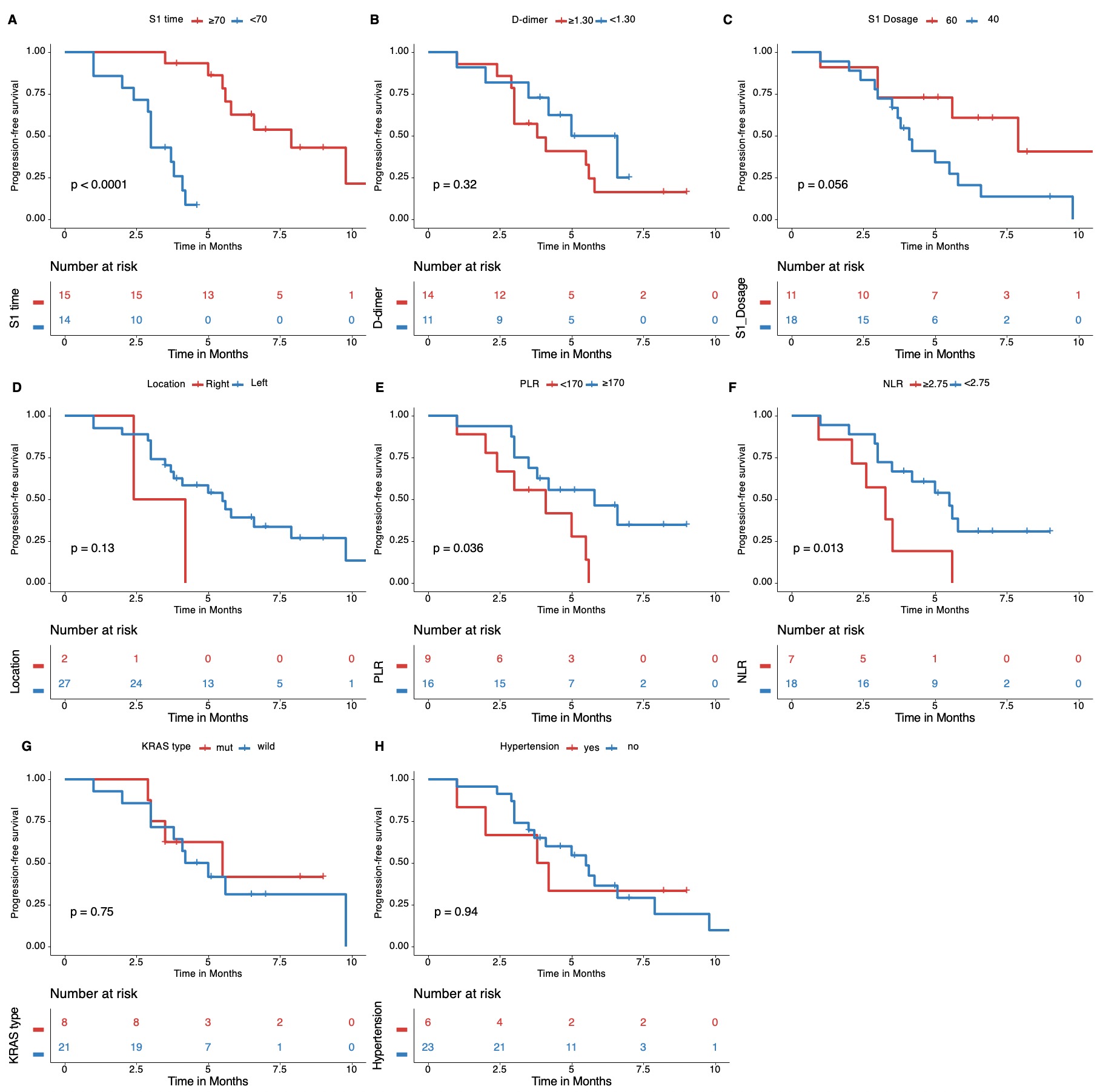

Supplement: Supplementary file 2 [file Image_1.jpeg]

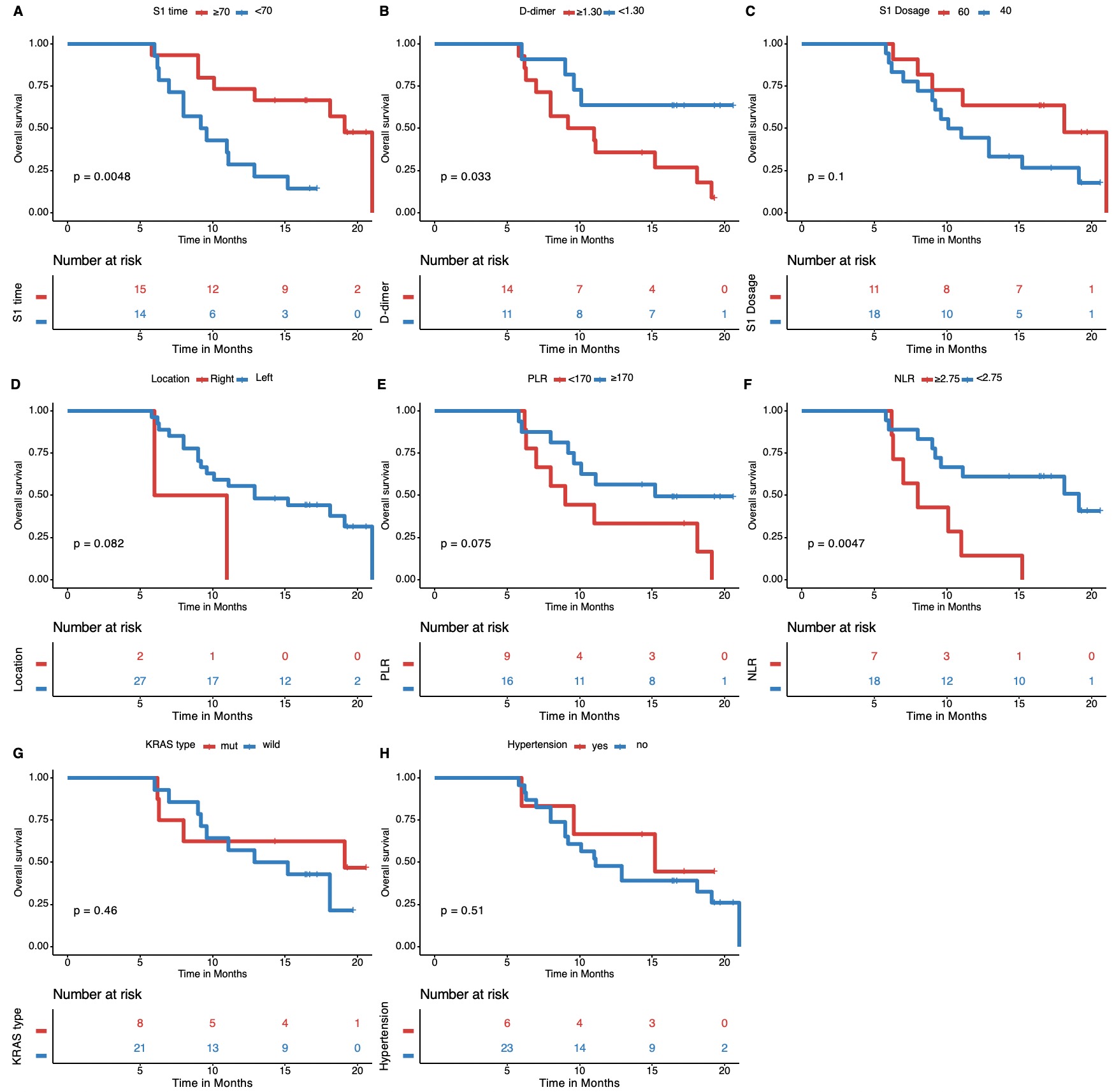

Supplement: Supplementary file 3 [file Image_2.jpeg]
